# A GEMPix‐based integrated system for measurements of 3D dose distributions in water for carbon ion scanning beam radiotherapy

**DOI:** 10.1002/mp.14119

**Published:** 2020-03-21

**Authors:** Johannes Leidner, Mario Ciocca, Andrea Mairani, Fabrizio Murtas, Marco Silari

**Affiliations:** ^1^ CERN 1211 Geneva 23 Switzerland; ^2^ Physics Institute 3B RWTH Aachen University 52074 Aachen Germany; ^3^ Fondazione CNAO 27100 Pavia Italy; ^4^ HIT 69120 Heidelberg Germany; ^5^ INFN‐LNF 00044 Frascati Italy

**Keywords:** gaseous detectors, GEMPix, ion beam radiotherapy

## Abstract

**Purpose:**

Commercially available systems for ion beam reference dosimetry in water are mainly based on ionization chambers. In those systems, a large number of small detectors are typically arranged in a two‐dimensional (2D) array or matrix to achieve high spatial resolution (order of several millimeters) and large field coverage at the same time. The goal of this work was to investigate the reliability of a detector of superior spatial resolution to perform three‐dimensional (3D) ionization measurements in carbon ion pencil beams.

**Methods:**

The GEMPix is a small gaseous detector with a highly pixelated readout, consisting of a drift region (with 2.8 cm^3^ × 2.8 cm^3^ × 0.3 cm^3^ volume), three gas electron multipliers (GEMs) for signal amplification and four Timepix ASICs with 55 µm pixel pitch and a total of 262,144 pixels. An integrated system was designed and built, which consists of a commercial water phantom with a three‐axis motorized arm, a reference large‐area ionization chamber for signal normalization to the beam output and the GEMPix itself. Measurements at different depths in water have been performed at the Italian National Centre for Oncological Hadrontherapy (CNAO) with three carbon ion beam energies. Lateral beam profiles measured with the GEMPix at the shallowest depth were compared to those measured with radiochromic EBT3 films in air in the position of the reference ionization chamber. The Timepix readout was calibrated in energy by using one independent depth scan with carbon ions of 150 mm range. Bragg peak curves were also simulated using the Monte Carlo FLUKA code as a reference.

**Results:**

Beam profiles measured with the GEMPix were smooth and showed similar shape and full width at half maximum when compared to those measured with radiochromic EBT3 films. Smooth, reproducible Bragg curves were obtained with statistical uncertainties of about 2%, matching FLUKA simulations of the Bragg curves within 15% for most data points. This difference is partially explained for the measurement with carbon ions of 150 mm range by a saturation effect in the GEMs. The high granularity of the readout allowed to produce 2D images of the deposited dose at different depths, as well as 3D data distributions.

**Conclusions:**

This paper demonstrates the capability of the GEMPix detector to measure the 3D dose distribution of carbon ions in water for a clinical pencil beam reliably. In the future, the detector area will be increased to cover fields of scanned beams. Measurements at higher beam intensities and with protons are planned.

## Graphical abstract

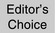

## 1 Introduction

The use of particle therapy to treat cancer is increasing and more than 200,000 patients have been treated in total with protons and carbon ions worldwide as of the end of 2018.^1^ The main advantage of particle therapy over photon radiation therapy is due to the so‐called inverted depth dose curve of charged hadrons (Bragg curve) that allows for highly conformal treatment plans with large dose gradients sparing better the normal tissue: the Bragg curve shows a relatively stable dose deposition in the entrance channel (plateau region), followed by an increasing dose deposition toward a maximum (the Bragg peak) close to the end of the range and a sharp distal dose falloff. For carbon ions there is a tail of dose deposition behind the Bragg peak caused by nuclear fragmentation. The dose is well confined in depth with a moderate lateral spread. Therefore, detectors for beam dosimetry and quality assurance should offer a good spatial resolution of better than 1 mm. For patient‐specific treatment plan verification, arrays of ionization chambers in a water phantom are often used.^2^ However, the spatial resolution is limited to the size of each ionization chamber, which is currently around 5 mm.

In this work, we describe the use of the GEMPix detector^3^ — a small gaseous detector with gas electron multipliers (GEMs^4^) and a 55 µm pitch pixelated readout — for measurements of the three‐dimensional (3D) dose distribution. A stand‐alone integrated system consisting of the GEMPix, a water phantom and a reference ionization chamber was developed. Two‐dimensional (2D) images at different positions in depth were acquired to calculate Bragg curves and 3D data distributions. Bragg curves were compared to FLUKA^5^, ^6^ Monte Carlo simulations of the integrated system. Preliminary measurements in a water phantom at the Italian National Centre for Oncological Hadrontherapy (CNAO)^7^ with the GEMPix, serving as a proof‐of‐principle but suffering from differences between GEMPix measurements and reference measurements, were reported in an earlier publication.^8^ The use of GEMs for measurements of the dose in hadron therapy has been described by other groups reading the signal of optical photons produced by scintillation in the gas, but with much lower spatial resolution than the results presented here (see e.g., Ref. [9, 10, 11]). Other detectors under development based on different technologies such as liquid scintillation^12^ also aim for a sub‐millimeter resolution and a 3D representation of the deposited dose.

## 2 Materials and Methods

### 2.A Experimental

**Fig. 1:**
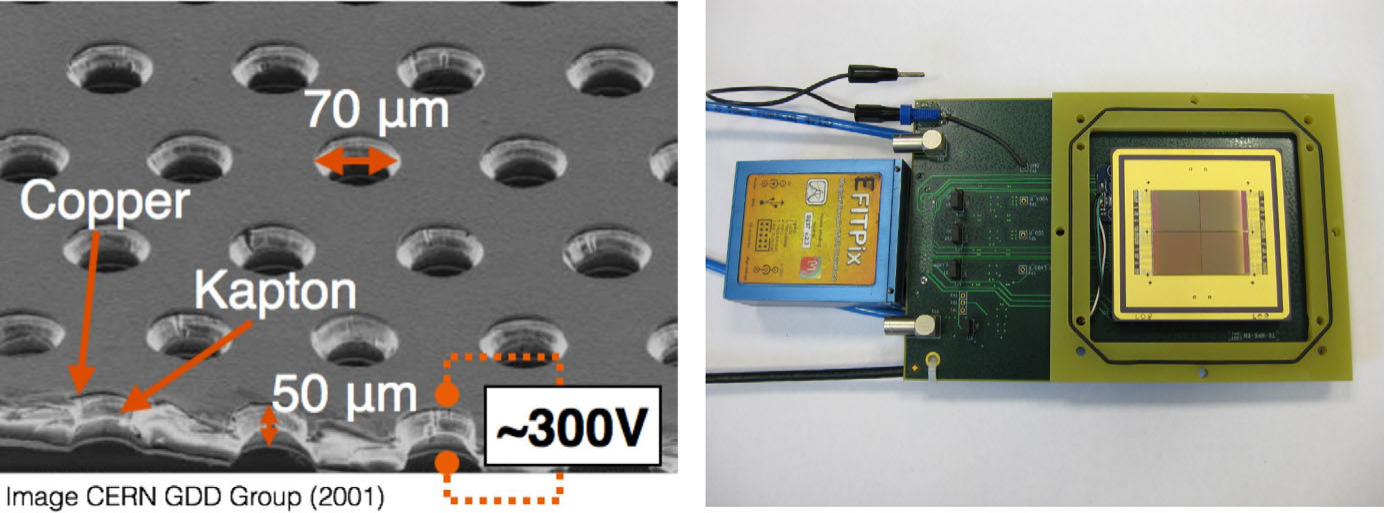
Image of a standard GEM foil (left, 14, 15) and of the opened GEMPix, showing the four Timepix ASICs (right). [Color figure can be viewed at wileyonlinelibrary.com]

**Fig. 2:**
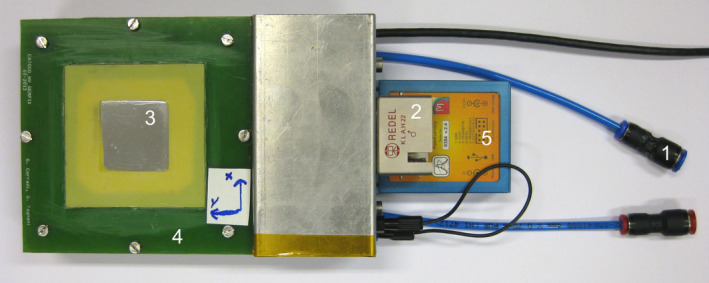
The GEMPix detector: (1) external gas supply, (2) external HV connector, (3) Mylar entrance window, (4) frame to hold the GEM foils, (5) FITPix readout. [Color figure can be viewed at wileyonlinelibrary.com]

The GEMPix is a small gaseous detector obtained by coupling two technologies developed at CERN, namely GEMs^4^ to four naked Timepix ASICs^13^ with 262,144 pixels of 55 μm^2 ^× 55 μm^2^ area for readout. Figure 1 shows images of a GEM foil and of the Timepix. Figure 2 shows the complete GEMPix detector. Ionizing particles produce electron‐ion pairs in a drift volume (2.8 cm^3^ × 2.8 cm^3 ^× 0.3 cm^3^). Electrons are then drifted toward a series of three standard GEMs and are multiplied in the holes of the GEM due to the applied high voltage of 330 V per GEM (approximately 66 kV/cm). Each GEM consists of a 50 µm thin Kapton foil serving as an insulator with 5 µm copper cladded on each side and shows a regular pattern of holes of 70 µm diameter and 140 µm distance between holes. Due to the thin Kapton layer, very large electric fields and therefore gains can be reached in the GEM holes. GEMs can be manufactured in various sizes and shapes and have found several applications in high‐energy particle physics and beyond. A relatively recent overview on GEMs can be found in Ref. [15]. In total, seven electric fields (drift, three GEMs, two transfer, and the induction field between the last GEM and the Timepix) are supplied in the GEMPix by a module specifically designed for this purpose (HVGEM^16^). A continuous flow of an Ar:CO_2_:CF_4_ (45:15:40 ratio) gas mixture is supplied externally at a rate of 5 l/h.

The electrons are detected by four naked Timepix ASICs which are usually coupled to a semiconductor sensor, but are used without this semiconductor sensor in the GEMPix, measuring directly the charge produced in the GEMs (hence the term “naked” Timepix). The Timepix ASICs are read out by the FITPix readout^17^ using the Pixelman software.^18^ The obtained quantity is the time over threshold (ToT) that is a measure for the deposited charge and therefore energy. The minimum operating threshold is 700 electrons. It was chosen slightly higher in this work such that the Timepix was almost noise free, that is, the signal without beam was negligible compared to that with beam. At 5.9 keV, the energy resolution is typically 20% (full width at half maximum [FWHM] divided by the mean value).^19^ By applying a correction for changes in ambient conditions such as temperature, the GEMPix was operated over 9 days with a stable response within ± 3%.^19^ A more detailed description of the GEMPix can be found in Refs. [3] and [19]. A triple‐GEM detector coupled to an earlier, coarser readout was compared to radiochromic film measurements for irradiation with protons and carbon ions in Ref. [20]. In that work, lateral beam profiles of the GEMPix for one carbon ion beam energy are compared to those obtained by radiochromic EBT3 films and found in very good agreement.

**Fig. 3:**
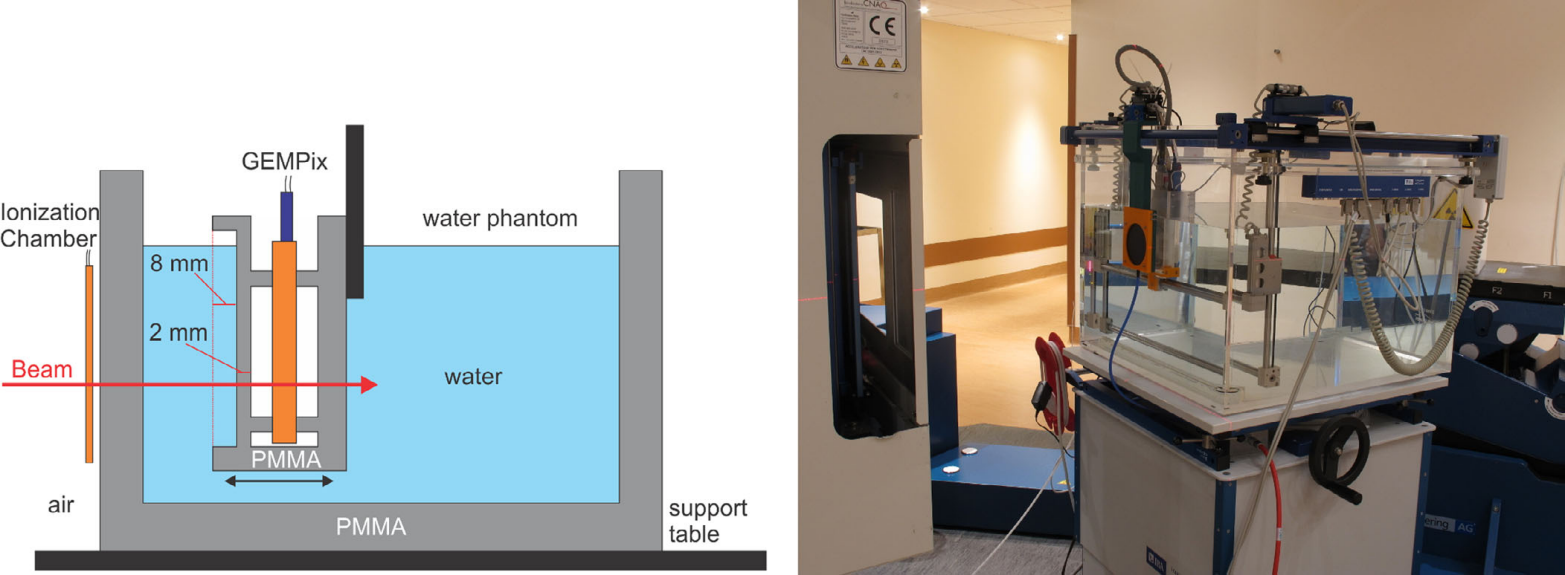
Schematic drawing (left, not to scale) and picture (right) of the setup. The beam enters from the left and passes through the reference ionization chamber before entering the water phantom. The GEMPix is inserted in a water tight box, which is mounted on the positioning system of the phantom. Therefore, depth scans are possible by remotely changing the distance between beam entrance window and GEMPix box. [Color figure can be viewed at wileyonlinelibrary.com]

The integrated system consists of a commercial water phantom, a commercial reference ionization chamber, the GEMPix, a trigger system, and other auxiliary equipment such as the high‐voltage supply, and the control and data acquisition software. The system can be setup relatively fast by keeping all equipment on trolleys. Figure 3 shows the system. An IBA Scanditronix Wellhöfer Blue Phantom type 2001 water phantom was used. It is equipped with a motorized positioning system on which the GEMPix is mounted. The maximum moving range is 48 cm in each direction with a resolution of 0.1 mm. An absolute calibration and correction of the positioning system via approaching and measuring the mechanical endpoints exists. More than 150 l of demineralized water is stored in a tank and pumped into the water phantom for the measurements.

A PTW model 34080 (PTW Freiburg, Germany) large‐area flat and thin ionization chamber (81 mm diameter, 72 mg/cm^2^ area density) was used as a reference detector, thus minimizing the perturbation of the particle beam. The ionization chamber was powered at +400 V and read out by the Pyramid Technical Consultants (PTC, USA) IC101. Ionization chamber and GEMPix were triggered externally with a common “start” pulse from an Arduino^21^ microcontroller to ensure synchronous data acquisition.

The software to control the integrated system is written in LabVIEW^22^ and enables semiautomatic data acquisition: settings including the duration of a single measurement, the number of measurements at each depth, and the exact choice of positions need to be adjusted before a depth scan. Then the actual scan is performed automatically.

Measurements were performed with carbon ions (C^6+^) at one of the fixed horizontal beam lines at CNAO, where a synchrotron delivers scanning proton and carbon ion beams to three treatment rooms. The smallest intensity characterized for clinical applications of 2*10^6^ ions per spill^23^ and three beam energies (280, 332, and 380 MeV/u resulting in ranges in water of 150 mm, 200 mm, and 250 mm, respectively) were used. GEMPix and reference ionization chamber measured simultaneously and each measurement lasted 10 ms.*The choice of a short measurement duration is guided solely by the fact that otherwise counters in the Timepix accounting for the ToT values will overflow. No dependence of the dose measurement on the exact choice of the measurement duration is expected, since each GEMPix measurement is normalized by the reference ionization chamber measurement of exactly the same duration. The measurement rate was limited during these measurements to about 1 Hz due to the trigger setup and the dead time of several 100 ms per measurement produced by the Timepix readout. Typically 60 measurements were taken at each depth position. A depth scan consists in 30–40 depth positions with smaller steps around the Bragg peak where the dose gradient is the largest. Measurements with calibrated radiochromic EBT3 films as used in standard quality assurance procedures at CNAO^23^ were performed in air at the isocenter (i.e., in the position of the reference ionization chamber) afterwards. The films were scanned with a resolution of 0.2 mm. One central spot (i.e., a single and undeflected pencil beam) was continuously delivered for each irradiation. A combination of two thin ripple filters (3 mm water equivalent thickness globally) was used to slightly enlarge the pristine Bragg peak width, according to CNAO standard clinical practice for carbon ion treatment plans.

### 2.B Monte Carlo simulation

**Fig. 4:**
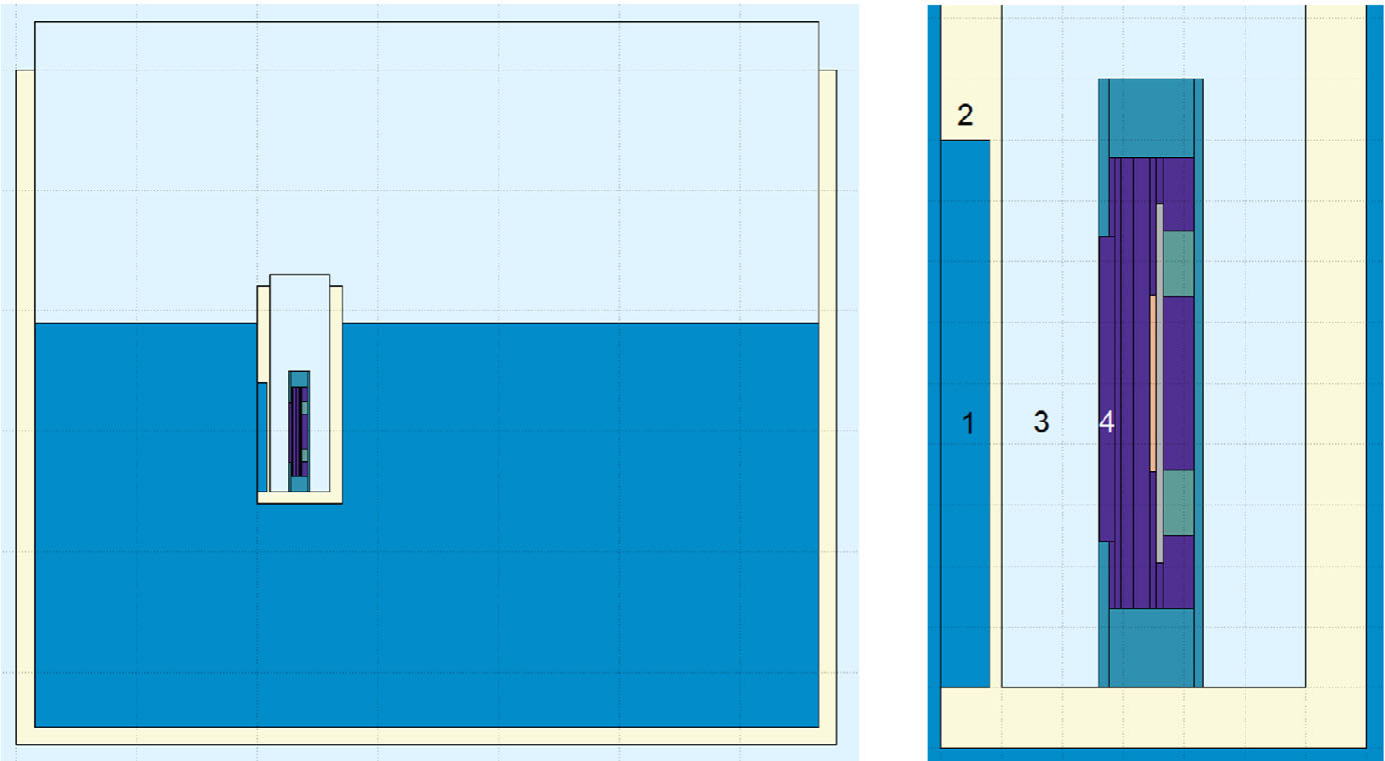
The implemented geometry in the FLUKA simulation: the water phantom with the GEMPix box (left) and a magnification of the GEMPix inside its box (right), showing the water (1), the PMMA box (2), the air in the box (3), and the GEMPix (4). [Color figure can be viewed at wileyonlinelibrary.com]

A FLUKA Monte Carlo simulation (version 2011.2x.2 with the physics model “HADROTHE”) was used for comparison with the measurements of the integrated system since other available measurement devices like the PTW Peakfinder^24^ have different sensitive areas. The simulation used in this work is based on the standard simulation for CNAO quality assurance^23^ by using phase space files generated at the beam exit window that contain the properties of the beam particles. Therefore, the beam characteristics such as spot dimensions are the same as in the standard simulation and they are similar to the measurements. Only from the beam exit window in the nozzle onwards, a dedicated simulation for this work was used. The same three beam energies as for the measurements were simulated and the simulation was stepwise tested: as a first step, results of the standard CNAO simulation were reproduced. Then the smaller sensitive area of the GEMPix was introduced. In a final step the geometry of the integrated system was implemented in a simplified design (Fig. 4). This design includes the main components such as the air in the treatment room, the phantom filled with water and the GEMPix box, but uses a simplified design of the GEMPix such that for example the GEMs are simplified to consist of a homogenous material without holes. No electric fields and detector response were simulated but the deposited dose in the drift volume was directly taken as the quantity of interest.

## 3 Results

**Fig. 5:**
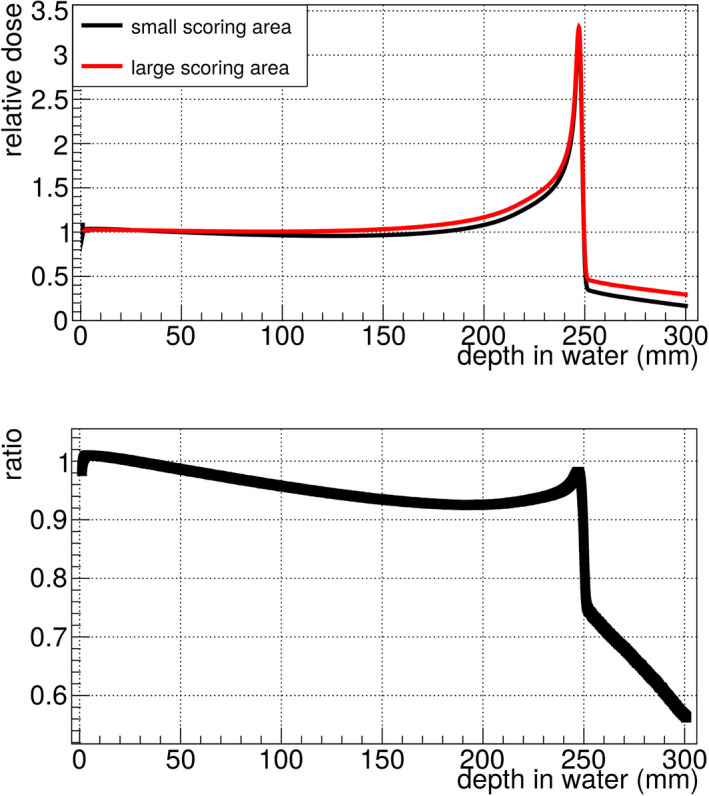
The simulated relative Bragg curve in water shows a clear dependence on the scoring area. When using the small scoring area (2.8 cm^2^ × 2.8 cm^2^, the area of the GEMPix), the dose is underestimated compared to the large area (50 cm^2^ × 50 cm^2^). Therefore, GEMPix measurements in this work were compared to the FLUKA simulation of the GEMPix (Bragg curves in Fig. 10) and not to the PTW Peakfinder with a larger area as in an earlier publication.^8^ [Color figure can be viewed at wileyonlinelibrary.com]

After reproducing the results of the standard CNAO FLUKA simulation with a scoring area of 50 cm^2^ × 50 cm^2^ in water, the smaller area of the GEMPix (2.8 cm^2^ × 2.8 cm^2^) was implemented. An underestimation of the dose in the GEMPix was found (Fig. 5). This underestimation increases with depth due to the growing beam size and is especially pronounced in the fragmentation tail. Therefore, GEMPix measurements could not be compared directly to other detectors such as the PTW Peakfinder since they have different areas. Instead, the GEMPix measurements are compared to the FLUKA simulation including the GEMPix design.

**Fig. 6:**
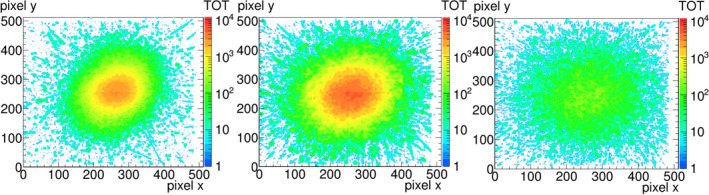
Typical 2D images obtained with the GEMPix in a carbon ion beam in the beam entrance region (left), Bragg peak (center), and fragmentation tail (right). X and y axes are the spatial coordinates labeled in pixel numbers, while the ToT value is color coded. The image size corresponds to the sensitive area of about 2.8 cm^2^ × 2.8 cm^2^. [Color figure can be viewed at wileyonlinelibrary.com]

**Fig. 7:**
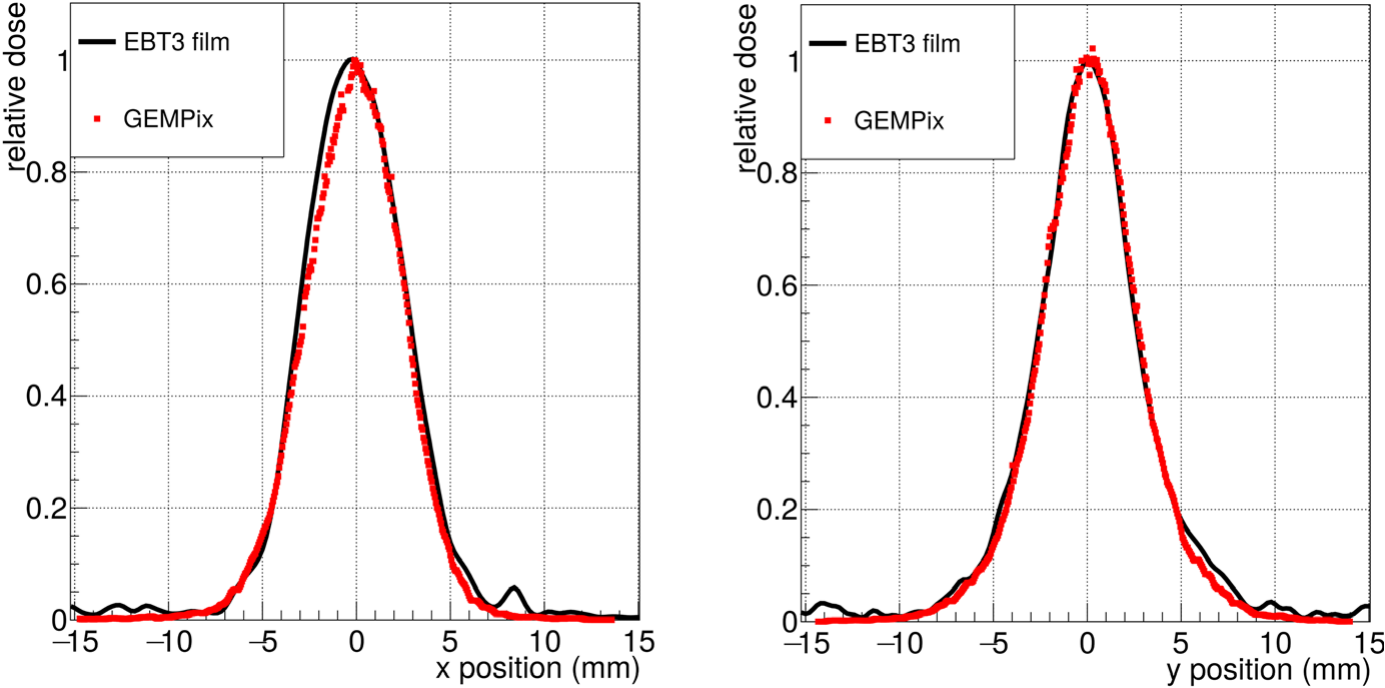
Comparison of transversal dose profiles for 150 mm range in horizontal direction (left) and vertical direction (right), measured with an EBT3 film (Ashland, Bridgewater, NJ, USA) and the GEMPix. In case of the GEMPix, these are the profiles of the 2D image shown in Fig. 6 (left, beam entrance region). [Color figure can be viewed at wileyonlinelibrary.com]

Measurements with the GEMPix produced 2D images of ToT values as shown in Fig. 6. Single particle tracks can be distinguished in the halo of the beam, while they superimpose in the beam center due to the integration over time. Relative horizontal and vertical beam profiles were calculated from the 2D image at the shallowest depth of 4 cm water equivalent thickness (the left image in Fig. 6). Figure 7 shows the comparison between GEMPix and radiochromic EBT3 film measurements for carbon ions of 150 mm range. The center value of the profile was set to one. The difference in position between points with a relative dose closest to 0.5 was taken as the FWHM with an uncertainty of 0.1 mm on each value: 5.9 mm (GEMPix, horizontal), 6.2 mm (EBT3, horizontal), 5.5 mm (GEMPix, vertical) and 5.4 mm (EBT3, vertical).

**Fig. 8:**
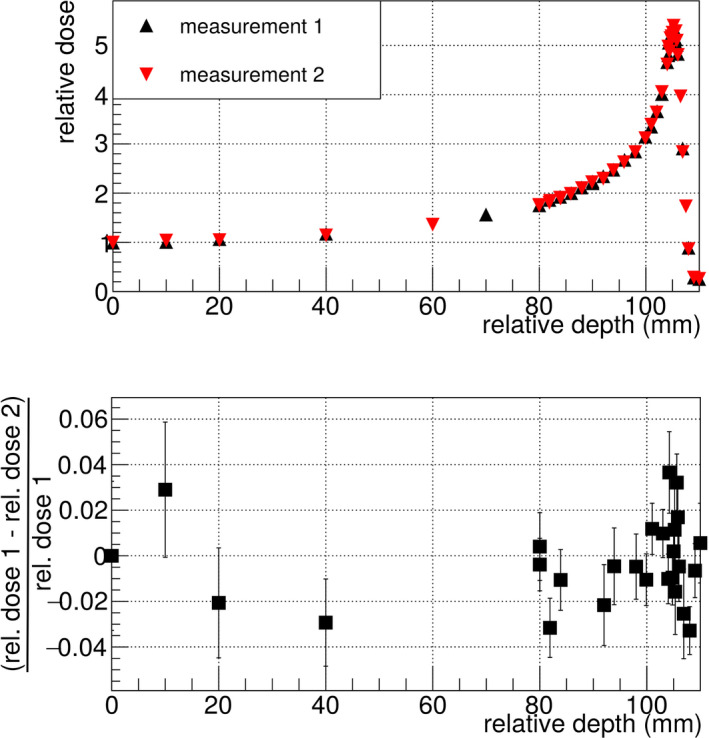
The measurement of the Bragg curve is well reproducible as repeated measurements are matching within the statistical uncertainties of typically around 2%. The upper plot shows the measurements (black and red points) and the lower plot shows the difference normalized to the first measurement. [Color figure can be viewed at wileyonlinelibrary.com]

The Bragg curve was calculated from the 2D images by summing over all pixel values in a single image, normalizing this value to the corresponding reference ionization chamber measurement, then averaging this normalized sum value over all measurements at the same depth and finally plotting this number vs depth. The variance of the mean value at each depth position was assigned as the statistical uncertainty and is typically around 2%. All Bragg curves in this work are shown relatively to the first point, which was set to one. The reliability of this procedure was tested by repeating a measurement with exactly the same settings twice, as shown in Fig. 8.

There is a gap between the readout ASICs of about 0.3 mm (visible in the GEMPix picture in Fig. 1), but the effect of this is hardly visible in the 2D images (Fig. 6) and smooth beam profiles were obtained without distortions close to the gap between ASICs (Fig. 7). When the lost dose in the gaps was estimated by assuming that there would be pixels in the gaps with the counts of the adjacent pixels, the effect of the gap on the relative Bragg curves was negligible compared to the uncertainties of about 2%.

**Fig. 9:**
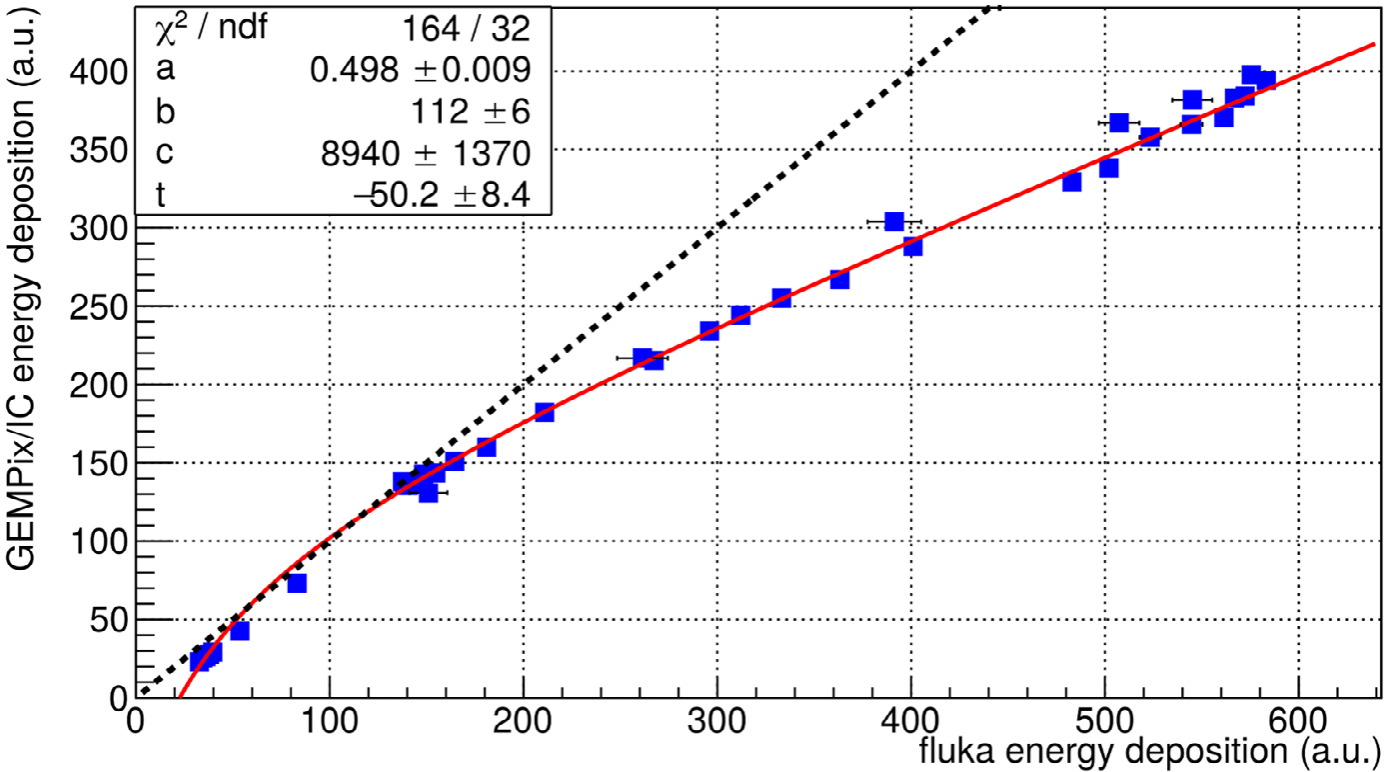
The fit of the standard surrogate function (red) to the data points (blue). The dashed black line shows the ideal response for a calibrated detector. Data presented here to develop the energy calibration were acquired in an independent depth scan with carbon ions of 150 mm range 1 month before the final measurements presented in Fig. 10 were performed. [Color figure can be viewed at wileyonlinelibrary.com]

The GEMPix measurements needed to be corrected by the Timepix energy calibration. The standard procedure for the energy calibration of the Timepix is described in Ref. [25]. However, this method cannot be applied directly to the GEMPix, since typically x rays depositing their energy in a single pixel are used for the Timepix calibration. However, x rays produce clusters in the GEMPix involving several pixels due to the diffusion in the gas. The best solution found for this work was to use an independent depth scan at CNAO for the calibration of all other measurements, that is, also for measurements at different beam energies than the calibration run. Measurements with the integrated system were plotted vs the “true” energy deposition, in this case the value simulated in FLUKA. Only an average value for the energy deposition at each depth was obtained. The fit of the standard surrogate function for the Timepix energy calibration was nevertheless accepted (Fig. 9). The fit function is:

$$f\left(x\right)=ax+b-\frac{c}{x-t}.$$

**Fig. 10:**
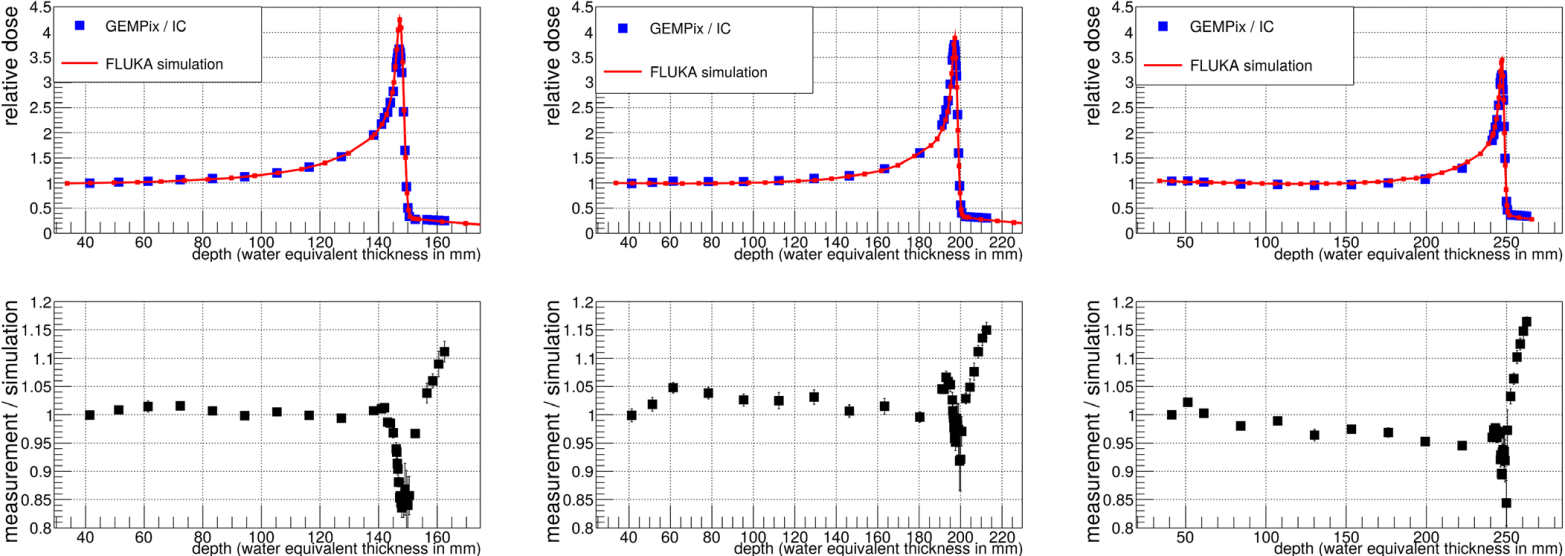
The upper plots show the Bragg curves for 150 mm (left), 200 mm (center), and 250 mm (right) range in water. The lower plots show the ratio of the experimental data to the FLUKA curve. Most data points match the simulation within ± 15%. Data were corrected for the energy calibration developed by another depth scan with carbon ions of 150 mm range taken 1 month before and presented in Fig. 9. [Color figure can be viewed at wileyonlinelibrary.com]

**Fig. 11:**
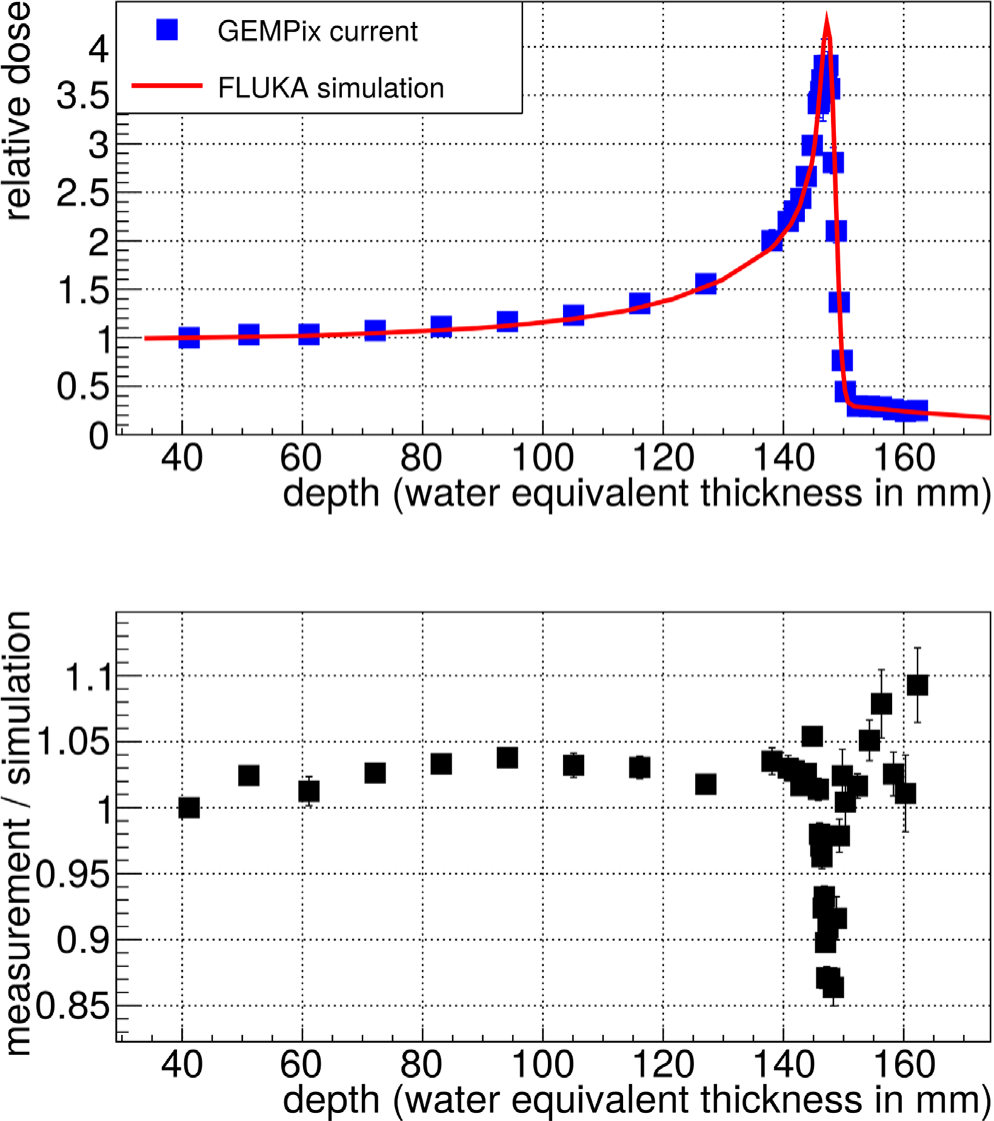
The current driven by GEM 3 is also a possible measurement of the Bragg curve (upper plot) and reveals saturation in the GEM 3 only in the Bragg peak region when compared to the FLUKA simulation. The lower plot shows the ratio between measurement and simulation. [Color figure can be viewed at wileyonlinelibrary.com]

Figure 10 shows the results for the three beam energies after the Timepix energy calibration. The obtained Bragg curves are smooth and match the FLUKA simulation with a maximum deviation of 15% for most of the data points. An underestimation of the dose of 15% was observed in the Bragg peak for 150 mm range (left plot in Fig. 10), which is partially explained by a saturation effect in the GEMs: it was possible to disentangle effects from the GEMs and from the Timepix readout by measuring the current driven by GEM 3 (the GEM foil closest to the Timepix readout) with the HVGEM module. In this way, a Bragg curve was obtained to check for saturation effects in the gas amplification process,^†^Note that only one current value integrated over the full size of the GEM was obtained. Therefore, this method can only be used for checks but does not produce for example the 2D images. without using the Timepix readout and therefore without any effect stemming from the readout. When scaling the GEM 3 current to the FLUKA simulation, a good match was obtained for the whole Bragg curve except for the Bragg peak, where a difference of up to 15% was noticed (Fig. 11). This is explained by a too large electron density in GEM 3 in the Bragg peak. The effect of GEM saturation due to this effect is described for example in Ref. [26]. As expected, this effect depends also on the number of electrons arriving at GEM 3 and was observed to become larger at higher gains when repeating the depth scans with three different GEM gains. The measurements at higher beam energies (center and right plots in Fig. 10) did not show such a pronounced saturation effect in the GEM current measurements since the absolute dose in the Bragg peak is lower for higher beam energies.^23^ A possible countermeasure to apply in the future is a lower GEM gain reducing the number of electrons at GEM 3, or a voltage unbalance over the three GEMs (i.e., the distribution of the voltage over the three GEMs is changed at constant voltage and therefore gain sum), an idea proven to have an effect in Ref. [27].

**Fig. 12:**
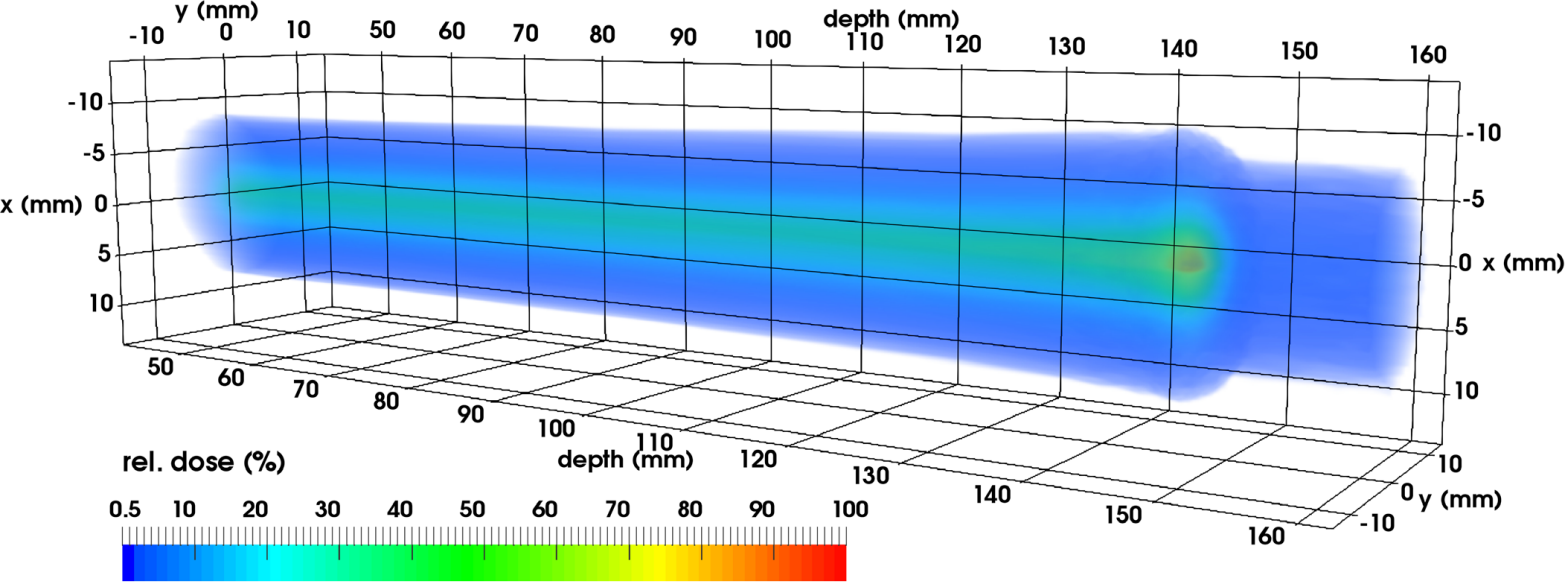
In this 3D representation of the dose, the carbon ion beam enters from the left. Lateral spread out, Bragg peak, and fragmentation tail are visible (color available online). [Color figure can be viewed at wileyonlinelibrary.com]

Figure 12 shows a 3D representation of the data, for which a linear interpolation between the measurements at different depths was used.

## 4 Discussion

In the following, the results obtained in this work are discussed also regarding the started upgrade to a larger detector area with a different readout technology^‡^Akkerman HB, Braccini S, Gallego Manzano L, et al. A Large Area GEMPix detector for treatment plan verification in hadron therapy. Abstract accepted for oral presentation at the “International Conference on Technology and Instrumentation in Particle Physics,” Whistler (Canada), 2020.
^,^ ^§^Akkerman HB, Braccini S, Gallego Manzano L, et al. A Large Area GEMPix detector for treatment plan verification in hadron therapy. Abstract submitted to the “9th Conference on New Developments in Photodetection,” Troyes (France), 2020.: this will not only allow to fully contain pencil beams in the sensitive area and enable measurements with scanned beams, but it is also expected to improve several other aspects discussed here.

Beam profiles measured with the GEMPix in this work were smooth and similar compared to those of radiochromic EBT3 films. The Bragg curves measured with the GEMPix as part of the integrated system, normalized to the carbon ion beam intensity with the PTW ionization chamber are smooth and reproducible within small statistical fluctuations of about 2%. A comparison to other detectors was not possible due to the different sensitive areas of the available ionization chambers. The difference in the size of the area is one reason why large differences were observed when comparing the GEMPix results directly with those of the PTW Peakfinder in a previous publication.^8^ A comparison to standard detectors such as the PTW Peakfinder will be performed again once the detector area is enlarged and the expected doses in the detectors are similar. In this work, the comparison with the FLUKA simulation shows differences smaller than 15% for most of the data points. This was achieved after correction by the Timepix energy calibration. The energy calibration is in general a standard procedure for the Timepix detector but needed to be adjusted to the GEMPix. With a different readout for the future large area detector, the energy calibration procedure will change. It is planned to calibrate the detector using radioactive sources or flat fields of hadron beams without the need of a Monte Carlo simulation. For the lowest beam energy, an underestimation in the Bragg peak of 15% was partially explained by a saturation effect in the GEMs. This effect can probably be reduced or even completely avoided in the future by applying a lower gain or a voltage unbalance in the three GEMs. This will be verified by linearity checks with the upgraded large area detector. It is expected that the saturation effect does not depend on the beam intensity as long as the electron clouds produced by carbon ions do not overlap, since the saturation effect depends on the energy density in a single GEM hole. GEMPix measurements with an integration time short enough that signals do not superimpose indicate that a beam intensity of 10^7^ ions per spill would still fulfil this criterion, but measurements will be needed in the future to prove this and check the behavior at even higher intensities to cover the full range of beam intensities typically used in clinical practice (on the order of 10^6^–10^8^ carbon ions per spill at CNAO).

The Bragg curve results are in agreement with those obtained by Seravalli et al.^10^ who detected the scintillation light of GEMs in clinical carbon beams and concluded that an observed 9% underestimation of the dose in the Bragg peak could be partly attributed to a saturation effect in the GEMs. The high voltage applied to the GEMs and therefore any possible saturation effect was smaller in their setup than in the present paper.

The GEMPix cannot only measure Bragg curves that are obtained by commercial detectors such as the PTW Peakfinder as part of a quality assurance procedure, but can also measure the dose distribution with a pixel pitch of 55 µm. Therefore, new types of information are acquired such as the 2D images or the 3D data representation, allowing to study for example the size of the beam vs depth.

## 5 Conclusions

An integrated system consisting of a commercial water phantom, a PTW ionization chamber and the GEMPix was setup and used for measurements of the 3D relative dose distributions of carbon ion beams at CNAO. The results in this paper represent a milestone on the way to a detector for quality assurance in hadron therapy. Measured Bragg curves at three beam energies match those simulated with FLUKA within ±15% for most data points. 2D images at each depth and a 3D data representation are obtained with the Timepix pixel pitch of 55 µm. The spatial resolution that is obtained in the 2D images with this pixel pitch still needs to be measured and compared to other high resolution detectors such as radiochromic films. Beam profiles match those of radiochromic EBT3 films.

In order to fully contain the dose of a pencil beam in the sensitive area, in the future the sensitive area of the GEMPix will be increased. Especially for quality assurance measurements of scanned beams, the detector needs to cover the full area of the radiation field with maximum about 20 cm^2^ × 20 cm^2^. About 200 Timepix ASICs would be needed to cover such an area and therefore, different readout options with a better scalability to larger sensitive areas are currently being investigated. Using a different readout, it could be possible to reduce the GEM gain and therefore potentially overcome the saturation observed in the current detector. This would also enable measurements at higher clinical beam intensities, for which the linearity of the new detector response will be checked.

This paper focuses solely on measurements with carbon ions but measurements with protons are foreseen with the large area detector, given the clinical interest. In the future, the imaging capabilities of the GEMPix could also be used to study better, for example, the fragments in the tail of the Bragg curve. Absolute dose measurements after calibration to reference ionization chambers are planned in the future.

## Conflict of Interests

The authors have no relevant conflict of interest to disclose.
